# Case of Spontaneous Destruction of the Alveoli, Second Bicuspis and First Molaris

**Published:** 1848-07

**Authors:** S. M. Sheppard

**Affiliations:** Petersburg, Va.


### ARTICLE II.

### *Case of Spontaneous Destruction of the Alveoli of the Second Bicuspis and First Molaris.* Reported by S. M. SHEPPARD, D. D. S., Petersburg, Va.

THE following case came under my observation in December, 1847, and, although I am unable to give a complete report of it, on account of its advanced state when first brought to my notice, still there may be facts connected with it worthy of record.

Mr. B., aged about 45, a farmer, consulted me in regard to an affection of the mouth, which first made its appearance about six months previous. I found, on examination, that the bicuspid and first superior molar, of the right side, had been removed, together with the gum and alveoli, up nearly upon a line with the termination of the roots of the teeth. The edges of the wound were somewhat reddish, not much inflamed, but no appearance of healing. I introduced a probe, which readily passed up into the antrum—the floor of that cavity having been entirely destroyed. The membrane of the cavity seemed to be in a healthy condition—judging from the fact, that no pain was occasioned by slight pressure upon it with the probe. The duct from the antrum to the nose was free, as was proved by forcing air through from one to the other; and fluids injected into the antrum, passed readily out at the nostril. To form a prognosis, I was dependent on the patient for a history of his own case up to this time. I was led to conclude from his physical condition, that the development of the affection of the mouth was the continuance rather than the beginning of disease. I learned, substantially, the following facts: About seventeen years ago, he had an attack of paralysis: from which, he only partially recovered. He was able, however, in some sort, to superintend his farm. His flesh wasted away, particularly from the left thigh and leg, leaving little else but skin and bone. He was a man of temperate habits; being an exemplary member of a christian church. He had a family also; a wife and four or five children. About six months previous to the time at which I was consulted, there was a feeling of tightness, and somewhat of numbness between the second superior bicuspid and first molar of the right side, which induced him to pick at it a good deal. It gave him little or no pain, and the gum had but little sensibility in it. In a few weeks the gum became hard and crust like, and began to slough; and as the alveoli became exposed they exfoliated. In a short time, by this process, the two teeth above named, became quite loose, and were removed. Hope was now entertained that the disease would be arrested; but it still continued without abatement until a large portion of the roots of the adjoining teeth were uncovered. Alum and other astringents were used without any beneficial effect. Very little pain was experienced up to this time. Being unable to discover any local cause for this affection, I supposed that local treatment would continue to be unavailing; and by my advice, he placed himself under the medical care of one of our most skilful surgeons. By the request of the surgeon I subsequently extracted three other teeth, viz. the first bicuspid, and the two remaining molares. He remained under treatment about two weeks, perhaps three, during which time I saw him occasionally. Finding himself rapidly declining, he returned home, and died in a few days. It may be proper to remark, that his teeth were all sound; that he had not lost more than one or two previous to the loss of those above referred to; that there were no dead roots of teeth in any part of his mouth; that the part affected had never received injury from a blow, or other accident, so far as recollected. Those last removed have nothing peculiar about them; and so far as their attachment remained, it appeared natural on removal from their sockets.

